# Supplementary material for: Population Genomics of the Maize Pathogen Ustilago maydis: Demographic History and Role of Virulence Clusters in Adaptation
Source: Genome Biol Evol. 2021 Apr 10;13(5):evab073. doi: 10.1093/gbe/evab073 (PMC8120014; doi:10.1093/gbe/evab073)
Supplement: evab073_Supplementary_Data [file evab073_supplementary_data.zip › SupplementaryFigureCaptions.docx]

**Supplementary figures**

Supplementary figure S1: Detailed results from the ADMIXTURE analysis, as represented by the PONG software. Each row represents the most likely population structure for a given number of subpopulations (K). Model fitting was repeated 10 times in each case with different initial random parameter values, and the most frequently inferred scenario was depicted. Ratios in blue indicate the number of replicates supporting the represented scenario in each case.

Supplementary figure S2: Distribution of F_ST_ values in 10 kb windows. The orange curve shows the fit of a two-normal mixture distribution.

Supplementary figure S3: Amplification of the *a* mating type locus. (A) Expected sizes in the reference isolates for the two idiomorphs *a1* and *a2*, the Mexican isolates B and C (having the Mexican *a1* and *a2* idiomorphs) and isolate A, predicted to carry both idiomorphs. Expected sizes of the Mexican isolates are based on the genome assemblies. (B) Amplification of the corresponding locus in isolates A (lane 1) and B (lane 2). M, size marker.

Supplementary figure S4: Sequencing coverage along scaffold 79 of the A isolate assembly. The region containing the *a1* idiomorph is depicted in red, the region containing the *a2* locus is plotted in green, and the central region of the scaffold (N) in blue. Coverage is measured in number of reads mapped to each position. (A) Per nucleotide coverage. Straight lines represent the linear regression on the corresponding data points. (B) Coverage distribution per region.

Supplementary figure S5: Phylogenetic tree of the mating type region of the b-locus. Left tree: *bW* alleles (*UMAG_00578*). Right tree: *bE* alleles (*UMAG_12052*). Nodes with a bootstrap value lower than 60% have been unresolved and displayed as multifurcations. Colors of the isolate names distinguish the ABCPQRSTUV and DEFGIKMNO subpopulations, as well as the three admixed individuals H, J, and L. Alleles of the reference strain 521 are is displayed in black, and all previously published alleles are in grey boxes.

Supplementary figure S6: Comparison of genetic diversity and rate of adaptive substitutions in clusters of effector genes, non-clustered effectors and non-clustered, non-effector genes after controlling for protein length. Legend as in Figure 5.
