# Supplementary figures and images for: Population Genomics of the Maize Pathogen Ustilago maydis: Demographic History and Role of Virulence Clusters in Adaptation
Source: Genome Biol Evol. 2021 Apr 10;13(5):evab073. doi: 10.1093/gbe/evab073 (PMC8120014; doi:10.1093/gbe/evab073)

K = 1

10/10 runs

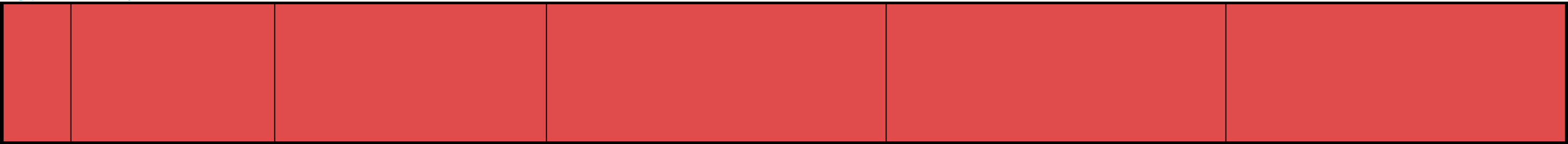

K = 2

10/10 runs

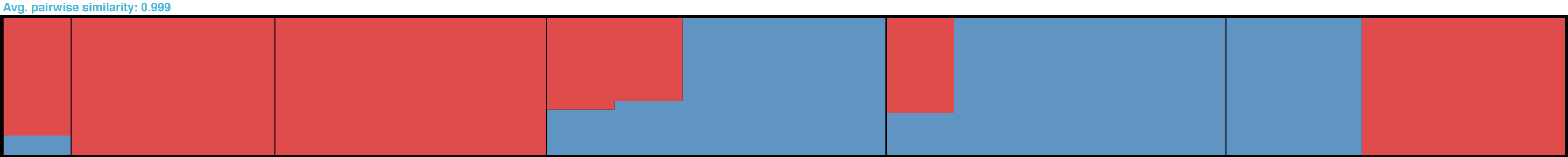

K = 3

4/10 runs

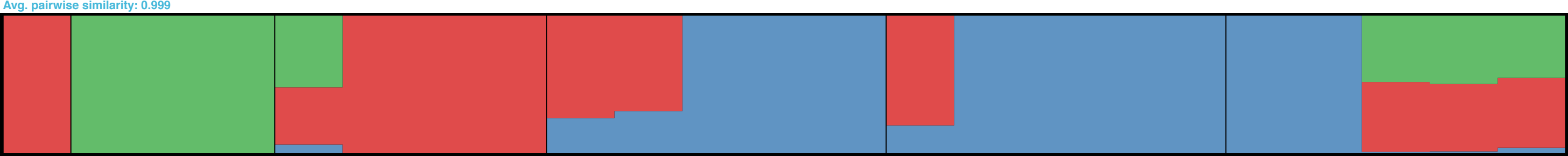

K = 4

3/10 runs

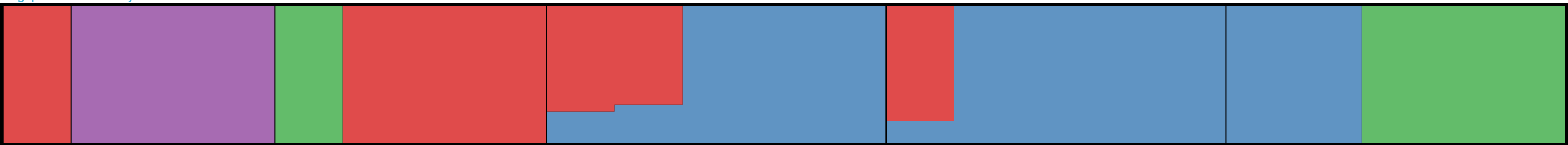

K = 5

6/10 runs

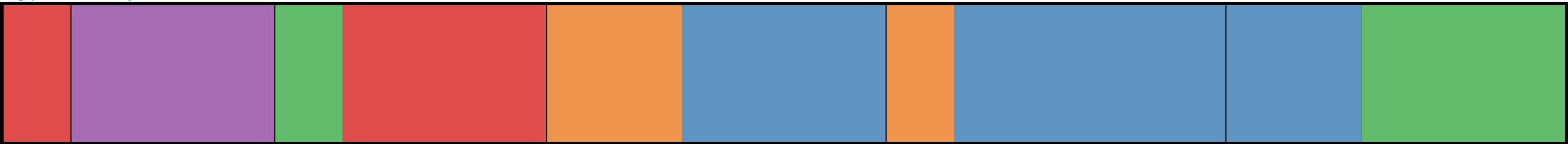

K = 6

6/10 runs

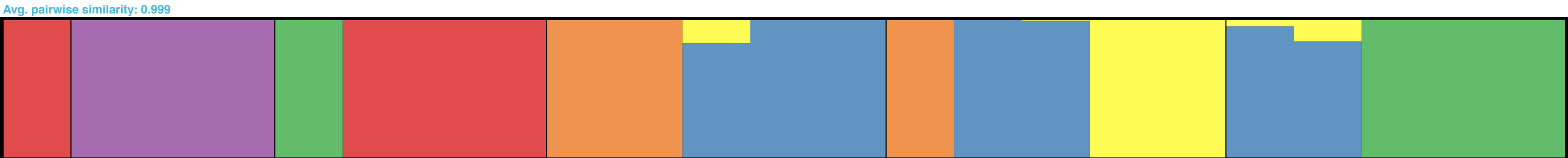

USA

Shelva

Toluca

Caraca

Padua

Irapuato

Supplement: evab073_Supplementary_Data [file evab073_supplementary_data.zip › FigureS1.pdf]

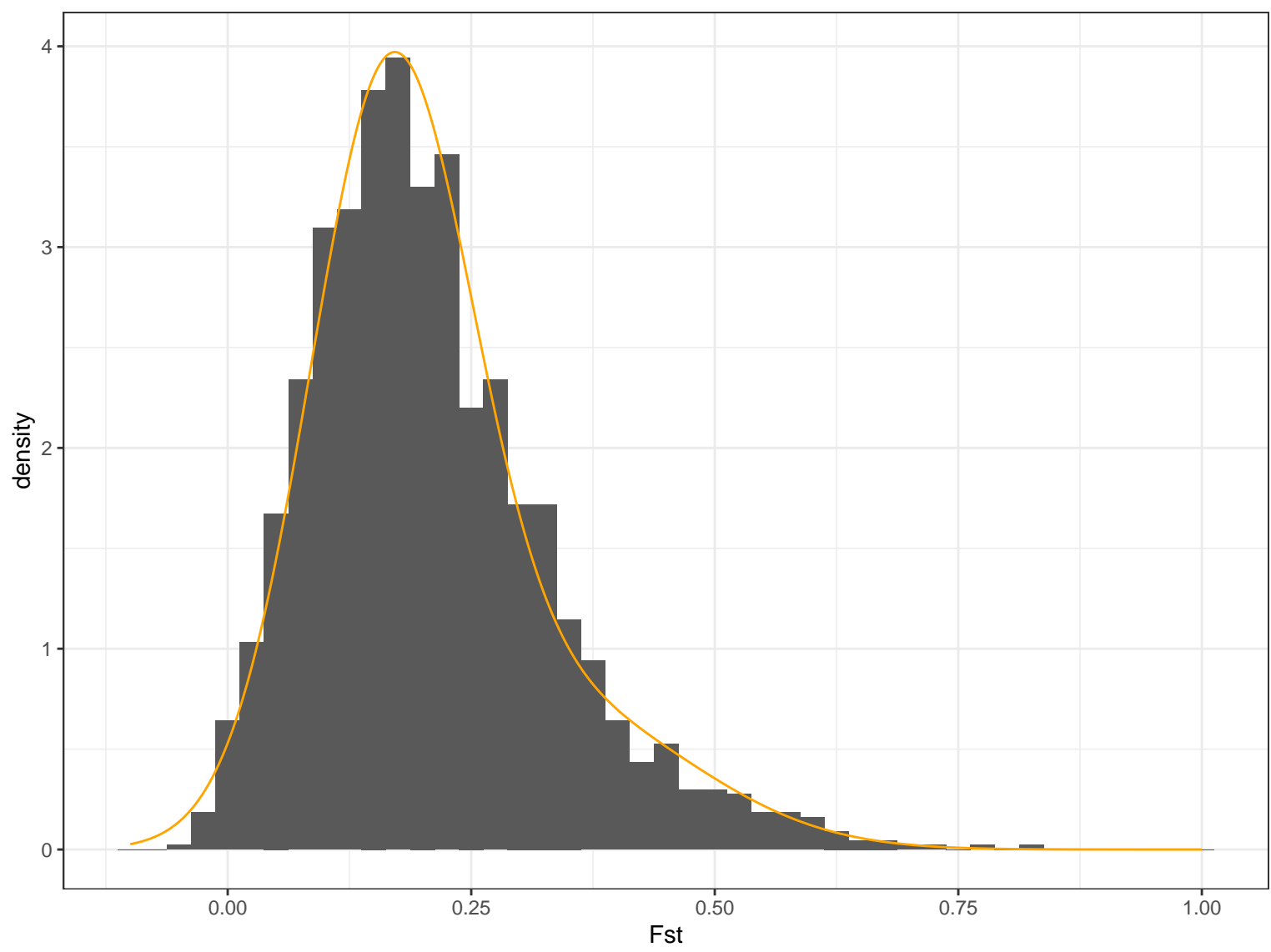

Supplement: evab073_Supplementary_Data [file evab073_supplementary_data.zip › FigureS2.pdf]

**A**

| Strain         | Expected size |
|----------------|---------------|
| Reference a1   | 4,961 bp      |
| A (a1 + a2)    | 11,890 bp     |
| B (Mexican a1) | 4,959 bp      |
| Reference a2   | 8,809 bp      |
| Mexican a2     | 7,082 bp      |

**B)**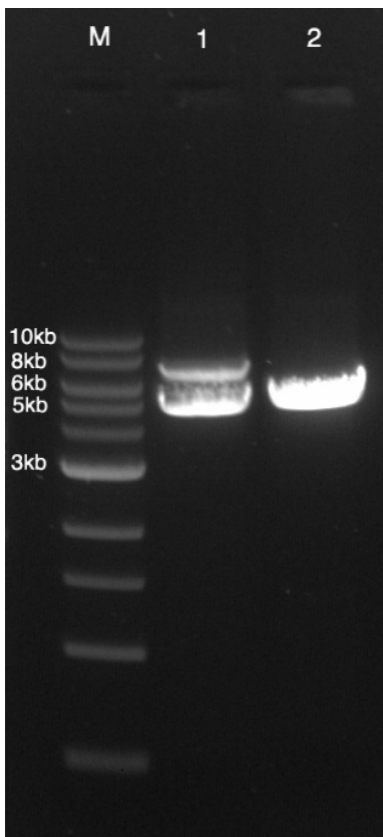

Supplement: evab073_Supplementary_Data [file evab073_supplementary_data.zip › FigureS3.pdf]

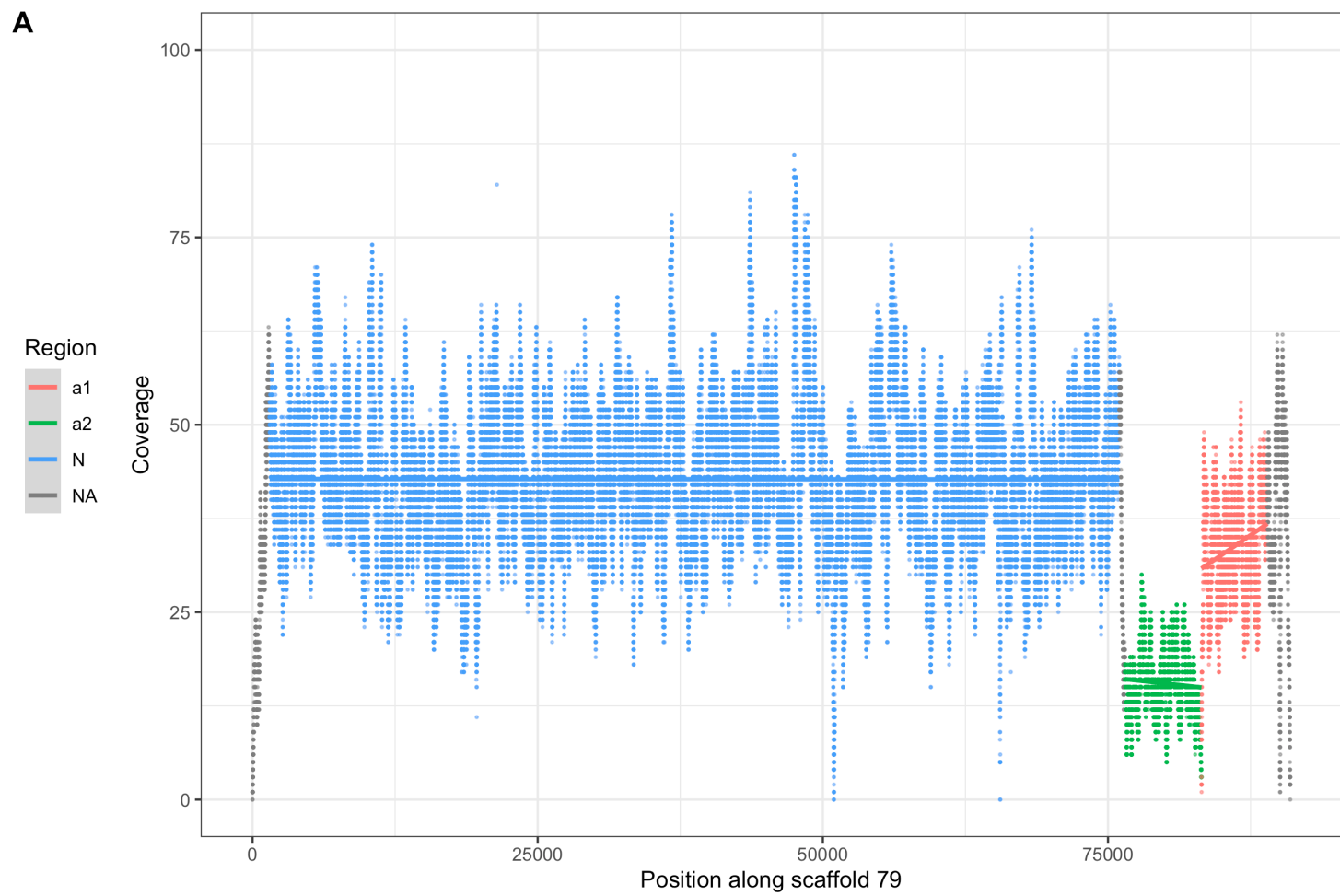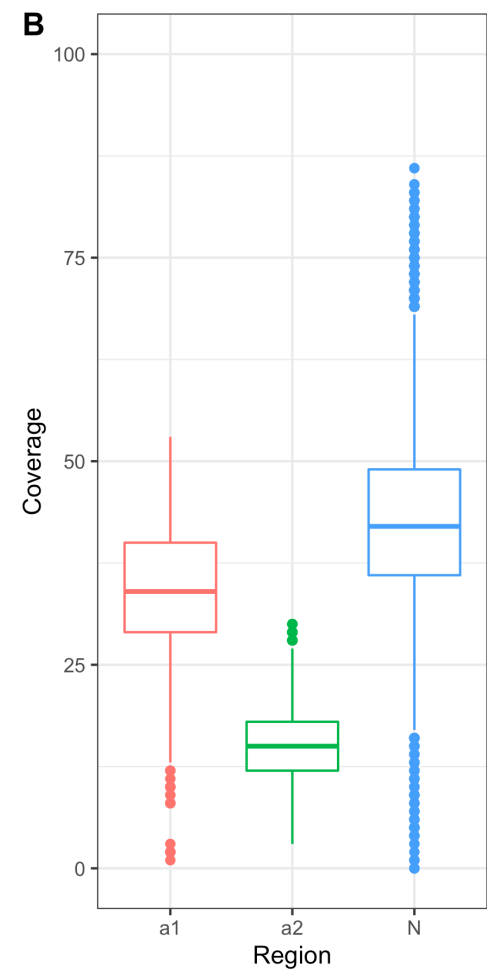

Supplement: evab073_Supplementary_Data [file evab073_supplementary_data.zip › FigureS4.pdf]

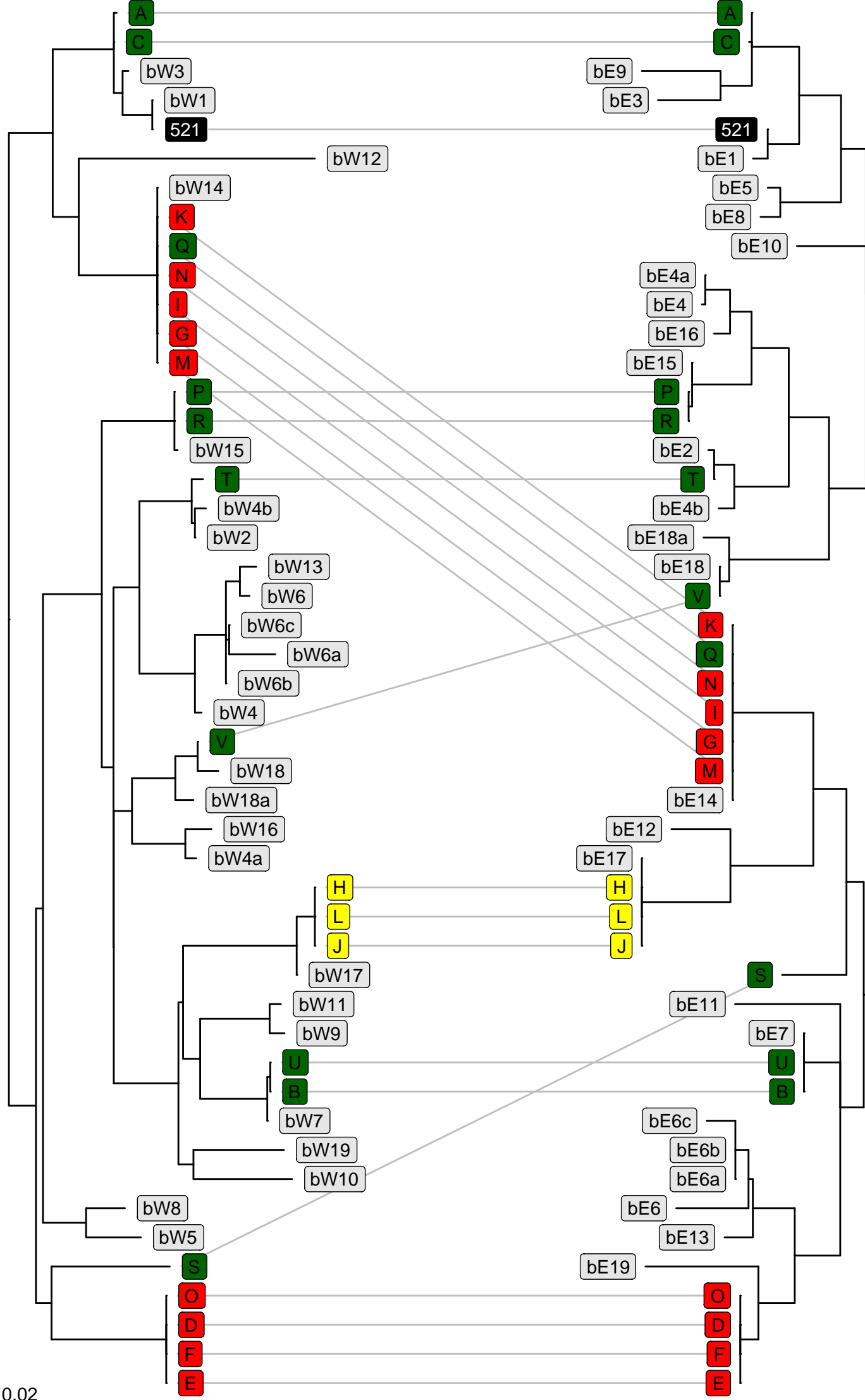

0.02

Supplement: evab073_Supplementary_Data [file evab073_supplementary_data.zip › FigureS5.pdf]

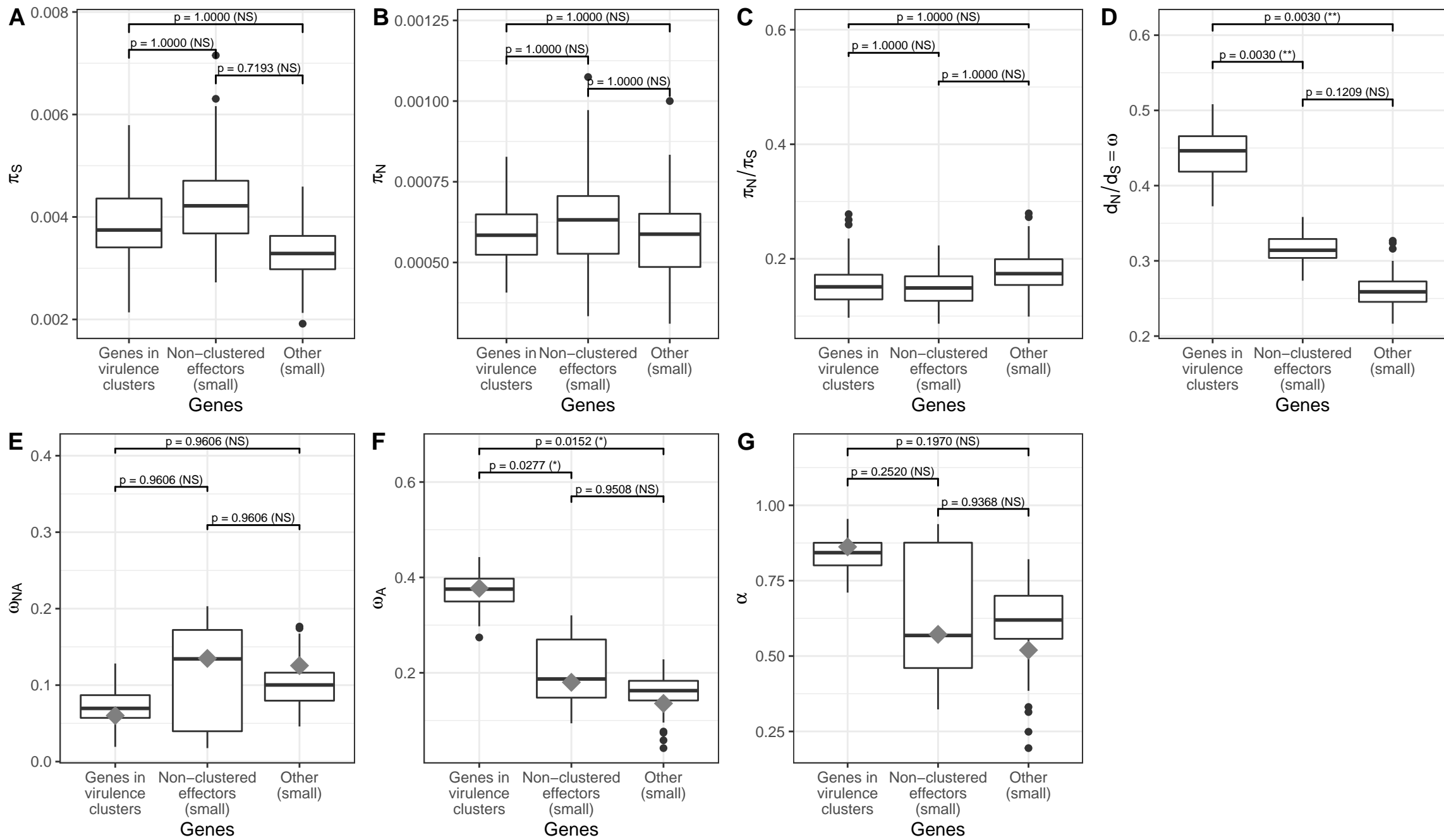

Supplement: evab073_Supplementary_Data [file evab073_supplementary_data.zip › FigureS6.pdf]
